# *Borrelia lusitaniae* and Green Lizards (*Lacerta viridis*), Karst Region, Slovakia

**DOI:** 10.3201/eid1212.060784

**Published:** 2006-12

**Authors:** Viktória Majláthová, Igor Majláth, Marketa Derdáková, Bronislava Víchová, Branislav Peťko

**Affiliations:** * Parasitological Institute of Slovak Academy of Sciences, Košice, Slovakia;; † University of P.J. Šafárik in Košice, Košice, Slovakia;; ‡ Institute of Zoology of Slovak Academy of Sciences, Košice, Slovakia

**Keywords:** *Borrelia lusitaniae*, *Lacerta viridis*, green lizard, *Ixodes ricinus*, Slovakia, lyme borreliosis, Karst, research

## Abstract

In Europe, spirochetes within the Borrelia burgdorferi sensu lato complex are transmitted by Ixodes ricinus ticks. Specific associations are described between reservoir hosts and individual genospecies. We focused on green lizard (Lacerta viridis) as a host for ticks and potential host for borreliae. In 2004 and 2005, a total of 146 green lizards infested by ticks were captured, and 469 I. ricinus ticks were removed. Borrelial infection was detected in 16.6% of ticks from lizards. Of 102 skin biopsy specimens collected from lizards, 18.6% tested positive. The most frequently detected genospecies was B. lusitaniae (77.9%–94.7%). More than 19% of questing I. ricinus collected in areas where lizards were sampled tested positive for borreliae. B. garinii was the dominant species, and B. lusitaniae represented 11.1%. The presence of B. lusitaniae in skin biopsy specimens and in ticks that had fed on green lizards implicates this species in the transmission cycle of B. lusitaniae.

## Abstract 2

TOC summary line: The green lizard is implicated in the transmission cycle of *B. lusitaniae.*

The causative agents of Lyme borreliosis, spirochetes of the Borrelia burgdorferi sensu lato complex, are maintained in natural foci by circulation between the vector ticks in the Ixodes ricinus complex and reservoir hosts of various vertebrate taxa. The B. burgdorferi s.l. complex encompasses 12 species (*1**–**3*); 4 species have been clearly established as pathogenic to humans: B. afzelii, B. garinii, B. burgdorferi s. s., and B. spielmanii (*4**–**6*). B. valaisiana and B. lusitaniae, which were previously considered nonpathogenic, may cause disease as well (*7**,**8*). Different species are associated with distinct ecologic features, levels of pathogenicity, and clinical symptoms in patients.

In Europe, I. ricinus ticks infest a wide variety of vertebrate hosts, such as mammals, birds, and lizards. The vertebrate hosts are necessary to maintain the tick population and may also serve as reservoirs for the pathogen. Therefore, the identification of reservoir host species is essential to clarify the transmission patterns of B. burgdorferi s.l. in natural foci. The importance of rodents for maintaining B. afzelii (*9*), and of birds for B. garinii and B. valaisiana (*10*), in endemic regions of Slovakia is now indisputable. The National Park Slovak Karst is within the region in which B. burgdorferi s.l. in questing ticks and birds has been reported (V. Tarageľová, unpub. data). In this area, 2 lizard species occur sympatrically, the common wall lizard (Podarcis muralis) and the green lizard (Lacerta viridis). The green lizard, the dominant species, is frequently infested by immature stages of I. ricinus ticks (*11*).

The importance of lizards in the maintenance cycles of B. burgdorferi s.l. spirochetes is still controversial. In Italy, B. lusitaniae was detected in blood and tissue samples of P. muralis (*12*). Furthermore, Psammodromus algirus, the most abundant lizard species in North Tunisia, was found to be the primary host for immature stages of I. ricinus. Thus, it could play a role in the circulation of borreliae (*13*). B. burgdorferi s.s., B. andersonii, and B. bisettii were detected in the blood of 9 lizard species in the southeastern United States (*14*). On the other hand, several other studies in the United States have shown that the lizards Sceloporus occidentalis and Elgaria multicarinata are reservoir-incompetent for borreliae because they possess borreliacidal factor in their blood (*15**,**16*). However, 2 lizard species, Eumeces inexpectatus and Anolis carolinensis, can sustain B. burgdorferi s. s. infection (*17*).

In the Slovak Karst (southeastern part of Slovakia), the green lizard is the major host for immature stages of I. ricinus ticks (*11*). Therefore, the main aim of this study was to find out whether green lizards can participate in the maintenance cycles of B. burgdorferi s.l. in natural foci and whether an association with specific borrelial genospecies exists.

## Materials and Methods

### Study Area

The study was conducted in the National Park Slovak Karst. This area represents a part of the Inner Carpathians in southeastern Slovakia (48°36´ N, 20°52´ E). The climate is warm with low humidity and average temperatures of -4°C in January and 18°C in July. The average rainfall is 700 mm/year.

### Tick and Lizard Collection

This survey was conducted in 2004–2005, from May to September, when lizards and ticks are active. Questing I. ricinus nymphs and adults were collected by flagging the vegetation in areas where lizards were sampled. Ticks were immediately stored in 70% ethanol.

Green lizards were captured along hiking paths by hand or by noosing, in which a loop made from fishing nylon was attached to the end of a wooden stick and dangled in front of a lizard, which would be captured as it walked through the loop. Animals were characterized by sex and age (adult, subadult, juvenile) and examined for ticks.

Ticks were removed with forceps immediately after capture and stored in 70% ethanol. Biopsy specimens (a 2-cm distal part of the tail and a 1-mm × 1.5-mm piece of skin from collar scales) were taken from each lizard with sterile scissors and put in separate vials with 70% ethanol. Ticks were identified to the species and sex. Only I. ricinus ticks were further examined for B. burgdorferi sensu lato.

### DNA Isolation

Immediately before extraction, ticks and tissues were dried for 30 min to evaporate the ethanol. Each sample was cut with a disposable sterile scalpel. Tissue DNA from lizards' tails and scales was extracted by using DNeasy tissue kit (Qiagen, Hilden, Germany). Extraction steps were conducted according to the manufacturer's protocol. Genomic DNA from ticks was isolated by alkaline hydrolysis (*18*). Incubation time was extended from 5 to 30 min. Isolated DNA was stored at -20°C.

### PCR

PCR amplification was performed in a 25-μL reaction mixture from the MasterTaq DNA polymerase kit (Eppendorf AG, Hamburg, Germany) containing 10.4 μL deionized water, 5 μL 5× TaqMaster PCR Enhancer, 2.5 μL 10× Taq buffer (with 15 mmol/L Mg^2+^), 1.5 μL 25-mmol/L solution of Mg (OAc)_2_, 0.1 μL Taq DNA polymerase (5 U/μL), 0.5 μL deoxynucleoside triphosphate (dNTP) mix (10 mmol/L) (Fermentas, Vilnius, Lithuania), 1.25 μL of each primer (10 pmol/μL) (Invitrogen, Paisley, Scotland), and 2.5 μL DNA template.

To verify that DNA had been successfully isolated from each tick, primers for the fragment of the tick's mitochondrial cytochrome b gene (620 bp) were used (*19*). Negative samples were excluded from the further analysis. Positive samples were examined for the presence of B. burgdorferi s. l. by amplifying a portion of the 5S (rrfA)-23S (rrlB) rDNA intergenic spacer (*20*). PCR products were subjected to electrophoresis on a 1% agarose gel, stained with ethidium bromide, and visualized with a UV transilluminator.

### RFLP Analysis

The positive PCR products of the 5S-23S rDNA intergenic spacer regions were further analyzed by restriction fragment length polymorphism (RFLP). Previously extracted DNA of B. afzelii, B. garinii, B. valaisiana, and B. burgdorferi s.s. were used as positive controls. For each positive sample, 13 μL amplified DNA was digested at 65°C overnight in a solution containing 5 U of Tru1I (300 U/mL) and 1× Buffer R (Fermentas). Electrophoresis was conducted in 16% polyacrylamide gel at 150 V for 3 h. The gels were stained with SYBR gold nucleic acid gel stain (Molecular Probes, Leiden, the Netherlands) for 20 min, and bands were visualized with a UV transilluminator. RFLP profiles that differed from the known profiles of positive controls were further analyzed by sequence analysis.

### DNA Sequencing of PCR Products

Sequencing was performed at the Department of Molecular Biology (Faculty of Natural Sciences Commenius University, Bratislava, Slovak Republic). PCR of the 5S-23S rDNA intergenic spacer was conducted according to the protocol described above. For the fla gene amplicons, DNA strands were sequenced as described previously (*21*). PCR products were purified by using a QIAquick PCR purification kit (Qiagen). The complementary strands of each sequenced product were manually assembled. Sequences were compared with GenBank entries by Blast N2.2.13 (*22*). Homologous sequences were aligned by using the CLUSTAL W Multiple Sequence Alignment Program (version 1.81) (*23*). Sequence similarity among the sequences were calculated by EMBOSS Align, a pairwise alignment algorithm (http://www.ebi.ac.uk/emboss/align).

The accession numbers of 5S-23S rDNA intergenic spacer sequences obtained in this study are DQ539339 and DQ539340. Accession numbers of flagellin sequences obtained in this study are DQ788618, DQ788619, and DQ788620.

### Data Analysis and Statistics

To estimate the probability of a tick's becoming infected after engorging on a green lizard and to measure the degree of infectiousness of infected animals, specific infectivity I_s_ (*24*) and transmission coeficient β_H-T_ (*9*) were calculated. Individual infectivity (i) is defined as the proportion of larvae derived from an individual lizard that are infected (i = l_i_/l_h,_ l_i_ is the number of larvae that become infected, and l_h_ is the total number of larvae derived from that host). The specific infectivity (I_s_) of a reservoir host species is defined as the sum of individual infectivities and number of individual lizards sampled (I_s_ = Σi_S_/n_S,_ n is the number of individual ticks captured). The host-to-tick transmission coefficient (β_H–T_) is defined as the portion of the sum of individual infectivities and the number of lizards that infected >1 larva (β_H–T_ = Σi_S_/n_iS_ (n_i_ is the number of individual hosts that gave rise to at least 1 infected tick). Differences in the prevalence of B. burgdorferi s.l. in I. ricinus were evaluated statistically with the 2-tailed χ^2^ test (degrees of freedom [df] = 1). A value of p<0.05 was considered statistically significant.

## Results

### Lizards and Infestation with Ticks

One hundred forty-six (84 male, 52 female, and 10 subadult) of 165 (89 male, 61 female, and 15 subadult) captured green lizards were infested by ticks during the study period. In total, 469 (199 larvae and 270 nymphs) ticks were removed and further identified as I. ricinus. Male lizards were infested with 363 ticks (131 larvae and 232 nymphs), which represented 77.4% of all collected ticks. Moreover, 53 tails and 102 skin biopsy specimens were taken from the captured lizards.

### B. burgdorferi Prevalence in Ticks Collected from Lizards

DNA isolation was successful in 464 ticks (197 larvae and 267 nymphs), from which the fragment of cytochrome b gene was amplified. These ticks were further analyzed for the presence of B. burgdorferi s.l. In total, 77 (16.6%) ticks carried borreliae. The infection prevalence between nymphs (15.2%) and larvae (17.6%) did not differ significantly (p = 0.49669, df = 1) (Table 1). Twenty-nine percent of tick-infested lizards carried >1 infected tick. Infected lizards yielded ≈2 infected larvae per host.

**Table 1:** Variability of *Borrelia burgdorferi* sensu lato in ticks collected on lizards

Stage	No. ticks examined	No positive ticks (%)	No. ticks positive for genospecies (% positive ticks)					
			*B. lusitaniae*	*B. afzelii*	*B. garinii*	*B. burgdorferi* s.s.	*B. valaisiana*	*B. lusitaniae* + *B. burgdorferi* s.s.
Larvae	197	30 (15.2)	26 (86.7)	1 (3.3)	1 (3.3)	1 (3.3)	1 (3.3)	0
Nymphs	267	47 (17.6)	34 (74.5)	5 (10.6)	1 (2.1)	2 (4.3)	0	5 (10.6)
Total	464	77 (16.6)	60 (77.9)	6 (7.8)	2 (2.6)	1 (1.3)	1 (1.3)	5 (6.5)

Genotyping with PCR-RFLP identified the following species: B. lusitaniae, B. afzelii, B. garinii,B. burgdorferi s.s., and B. valaisiana. Of the B. burgdorferi–positive ticks, most (77.9%) were infected with B. lusitaniae. The presence of this species was significantly higher than that of other species (p<0.001). B. lusitaniae was detected in 26 (86.7%) larvae. B. afzelii, B. garinii, and B. burgdorferi s. s. each were found in 1 larva. Of the 47 B. burgdorferi–infected nymphs, 34 (72.3%) were infected with B. lusitaniae, 5 (10.6%) with B. afzelii, 2 (4.3%) with B. burgdorferi s. s., and 1 (2.1%) with B. garinii. A mixed infection of B. lusitaniae and B. burgdorferi sensu stricto was found in 5 (10.6%) nymphs (Table 1). Nymphs and larvae did not differ significantly in the prevalence of B. lusitaniae.

Male lizards were parasitized by 61 (79.2%) of 77 infected ticks. Variability of detected genospecies was higher in ticks collected from male than from female lizards. Larvae that fed on female lizards were only infected with B. lusitaniae. Out of 7 infected nymphs collected from females, B. lusitaniae was present in 5 and B. afzelii in 1 tick; B. lusitaniae and B. burgdorferi s.s. were detected as mixed infection in 1 nymph. The specific infectivity from lizards to larval ticks was highest for B. lusitaniae. The specific infectivity of female lizards was slightly higher than that of males (Table 2).

**Table 2:** Specific infectivity (I_s_) and host-to-tick transmission coefficient (β_H-T_)

Genospecies	Males		Females		Total	
	I_s_	β_H–T_	I_s_	β_H–T_	I_s_	β_H–T_
*Borrelia burgdorferi* s.l.	0.0477	0.605	0.0221	0.571	0.0697	0.5753
*B. lusitaniae*	0.0377	0.518	0.0221	0.571	0.0597	0.5263
*B. garinii*	0.0012	0.01	–	–	0.0012	0.01
*B. afzelii*	0.0012	0.01	–	–	0.0012	0.01
*B. burgdorferi* s.s.	0.0012	0.01	–	–	0.0012	0.01

### B. burgdorferi Prevalence in Lizards

Isolated genomic DNA from tails and skin biopsy specimens from collar scales was tested for the presence of B. burgdorferi sensu lato. None of 53 tested tail samples was positive. Of 102 skin biopsy specimens collected from green lizards, 19 (18.6%) tested positive. Differences in infection prevalence between sexes (18.2% in males vs. 23.7% in females) were not significant. Of 9 skin biopsy specimens from subadult individual lizards, 2 (22.2%) were borrelia positive. The most frequently detected genospecies was B. lusitaniae (94.7%), which was present in 18 samples. One lizard was infected with B. afzelii.

### B. burgdorferi Prevalence in Questing Ticks

Cytochrome b was amplified in 325 of 331 (71 female, 73 male, and 187 nymph) questing ticks. Therefore, only these 325 ticks (71 female, 71 male, and 183 nymph) were analyzed further for the presence of B. burgdorferi s.l. Sixty-three (19.3%) ticks tested positive. B. burgdorferi prevalence in female ticks was the same as in male ticks (22.5%), and it was lower in nymphs (16.9%).

RFLP analysis of the amplified products resulted in 5 distinct profiles. Of the 63 positive ticks, 21 (33.3%) were infected with B. garinii, 19 (30.2%) were infected with B. afzelii, 8 (12.7%) were infected with B. burgdorferi s. s., 7 (11.1%) were infected with B. lusitaniae, and 7 (11.1%) were infected with B. valaisiana. One nymph was infected simultaneously with B. garinii and B. valaisiana (Table 3).

**Table 3:** Variability of *Borrelia burgdorferi* sensu lato in questing ticks

Stage	No. ticks examined	No. positive ticks (%)	No. ticks positive for genospecies (% of positive ticks)					
			*B. afzelii*	*B. garinii*	*B. valaisiana*	*B. burgdorferi* s.s.	*B. lusitaniae*	*B. valaisiana + B. garinii*
Nymphs	183	31 (19.6)	13 (41.9)	12 (38.7)	2 (6.4)	3 (9.6)	0 (0)	1 (3.2)
Females	71	16 (22.5)	4 (25)	4 (25)	3 (18.7)	3 (18.7)	2 (12.5)	0 (0)
Males	71	16 (22.5)	2 (12.5)	5 (31.3)	2 (12.5)	2 (12.5)	5 (31.3)	0 (0)
Total	325	63 (19.3)	19 (30.2)	21 (33.3)	7 (11.1)	8 (12.6)	7 (11.1)	1 (1.5)

### Sequence Analysis

Representative samples of RFLP profiles that were different from the known profiles of positive controls were sequenced. The fragment of the 5S-23S rDNA intergenic spacer obtained from the B. burgdorferi s.l.–positive nymph (538N) from lizard belonged to B. lusitaniae. It was 100% identical to a Borrelia-positive skin biopsy specimen (277S) sampled from a lizard. Both obtained sequences were 100% identical with the Turkish B. lusitaniae strain Tr213 (AB 091802) and 98.9%, 98.4%, and 94.5% similar to PotiBL37 (AY 463167), PotiB2 (L30131), and PotiB3 (L30132) strains from Portugal, respectively. To better characterize B. lusitaniae circulating in ticks and lizards from Slovak Karst, the fla gene from a B. burgdorferi s. l.–positive questing adult tick and skin biopsy specimen from collar scale was amplified and sequenced. The flagellin sequence of B. lusitaniae detected in a skin biopsy specimen (277 S) was 100% identical and 99.6% similar to B. lusitaniae detected in questing adult ticks (43 ZLIF, 47 ZMLIM), respectively. Genotypes 277S and 43ZLIF were 100% identical with the Turkish B. lusitaniae strain Tr213 (AB091812) as well as with the Polish strain D23–04 (DQ 016623). Genotype 47ZLIM was 99.6%, 99.6%, and 99.4% similar to Tr213, D23–04, and PotiB2 (DQ111036), respectively.

## Discussion

The role of lizard species in maintaining B. burgdorferi s.l. has not been clearly elucidated yet. In United States, some lizard species have sustained borrelial infection (*14**,**17*); however, other species are incompetent reservoir hosts (*15**,**16*). The reservoir competence of lizards seems to be species specific. Therefore the aim of our study was to establish whether a relationship exists between green lizards, the dominant lizard species in the Slovak Karst, and B. burgdorferi s.l., which circulates in this area.

Seventeen percent of ticks that fed on lizards were infected with B. burgdorferi s.l. Seventy-eight percent of all infected ticks were infected with B. lusitaniae. Moreover, 18.6% of skin biopsy specimens from lizards were positive for B. burgdorferi s.l., and almost all (94.7%) were infected with B. lusitaniae. Similarly, B. lusitaniae have been detected in blood and tissue samples of Podarcis muralis in Tuscany in Italy, where borreliae were detected in 2 of 14 tested whole tails from lizards (*12*). At the beginning of our study, we also collected the distal tip of a lizard's tail because this method is minimally invasive and convenient for obtaining a tissue sample. The tissue at the tail, however, is squamous and keratinized, and none of the collected samples was borreliae positive. Therefore, we also obtained skin biopsy specimens from collar scales. These are elongated and extend from the skin on the ventral side, so collecting them is minimally invasive and perhaps more likely to detect infection with B. burgdorferi s.l. because most of the immature I. ricinus ticks parasitize at the dorsal area (pers. observation). Furthermore, collar scales were chosen to avoid detecting the borreliae that persist in the skin after feeding of the infected ticks, which may enable infection of ticks by "extended co-feeding" (*25*). In this manner, incompetent host species may contribute to the circulation of B. burgdorferi s.l. in nature. For example, in England, I. ricinus ticks cofeeding on sheep become infected with B. burgdorferi, although sheep themselves are refractory to infection (*26*). In Europe the principal importance of cofeeding to Lyme disease ecology has been suggested to be the extent of the range of vertebrate host species that contribute significantly to the maintenance of B, burgdorferi s.l. spirochetes in nature (*27*). Therefore, cofeeding transmission could also be responsible for B. afzelii, B. garinii, and B. burgdorferi s.s. infection in larvae that fed on lizards collected in our study, even though skin biopsy results yielded mostly B. lusitaniae. Another possible explanation for the presence of non–B. lusitaniae spirochetes is that these larvae may have been infected transovarially (*28*). Cofeeding transmission might explain why individual lizards with borreliae negative skin biopsy specimens carry borreliae-positive larvae. Because the quantity of borreliae is low in the vertebrate host and may lodge in deeper organs, detecting them in skin biopsy specimens may not always be possible (*29*). Thus, a negative skin biopsy result does not prove conclusively that the lizard is not infected.

Despite the fact that male lizards hosted >75% of all host-feeding ticks, as well as 79.2% of all infected ticks, the specific infectivity and host-to-tick transmission coefficient were almost the same for male and female lizards. The seasonal activity of green lizards and different patterns in male and female behavior were monitored in the Slovak Karst (I. Majlath, unpub. data). Larger numbers of ticks feeding on male lizards are associated with higher male activity in spring months, when tick activity peaks as well. Male lizards end hibernation first and are active when the air temperature reaches 10°C–12°C. They need to restock the energy that was depleted during winter and to gain energy for fighting other male lizards to compete for territory and females, for seeking female lizards, and for mating. Female activity increases in summer months when they are incubating eggs.

As determined by PCR, the overall prevalence of infection in our sample of questing ticks (19.3%) is consistent with 20.5% found in southern Czech Republic (*20*) but lower than that reported for a geographically close area in western Slovakia (40%–49%) (*30*). The total prevalence was higher in adults (22.5%) than in nymphs (19.6%), which is in agreement with the general pattern of increasing Borrelia prevalence through the life stages of ticks as their adults feed on a multiple hosts (*31*). The total prevalence of borreliae in male and female ticks was identical, but the distribution of genospecies was different. B. garinii was the predominant genospecies in this locality. B. garinii and B. valaisiana are the most commonly reported species in central Europe (*32*).

The high prevalence of B. lusitaniae in borrelia-positive larvae and nymphs as well as skin biopsy specimens from lizards suggests that green lizards are susceptible and transmission competent for B. lusitaniae. On the other hand, a lack or low prevalence of other genospecies in ticks that had fed on lizards may suggest that these genospecies could be negatively selected against by green lizards. A similar suppressive effect of Madeiran wall lizard (Podarcis dugesii) on the transmission of spirochetes was observed (*33*). Borreliacidal activity against B. burgdorferi s.s. was observed in the lizards S. occidentalis and E. multicarinata in North America (*15**,**16*). These findings add to the growing support for the hypothesis that there are Borrelia species-specific associations with specific reservoir host species that result from Borrelia species–specific interactions with host serum complement (*29*).

Significant differences were found in B. lusitaniae prevalence in fed larvae compared with questing nymphs (p<0.001, df = 1); none of 183 examined nymphs was infected by this genospecies. This finding raises the questions of whether borreliae are eliminated during molting and thus do not contribute to the transmission cycle or whether we were just unable to detect it. Significant differences were found in B. lusitaniae prevalence also in fed nymphs compared with questing adults (p<0.01, df = 1). The infection prevalence decreased from 74.5% in fed nymphs to 5% in questing adults. Reduction of infection prevalence has been observed in B. afzelii from 47% in nymphs engorged on the rodents to 7% in questing nymphs (*9*).

The occurrence of B. lusitaniae in ticks is frequent in some areas of the Iberian Peninsula and North Africa, where the organism often represents the only species of B. burgdorferi s.l. complex (*13**,**34*). In the rest of the Europe, it has been isolated or detected less frequently, with low prevalence in ticks (*30**,**35**,**36*). The prevalence of B. lusitaniae is the highest in southern Europe and can be exported to other areas by hosts such as birds (*37*). The 5S-23S rDNA and flagellin sequences of B. lusitaniae–positive ticks and skin biopsy specimens in our study were 100% identical to the B. lusitaniae strain Tr213 from a tick in Turkey (*38*). The distribution of this borrelial species may be associated with the distribution range of reservoir hosts, including lizards, that inhabit drier and warmer areas. These ecosystems are less abundant in central Europe than in the Mediterranean. Thus, lizards may influence the transmission cycle of borreliae in some localities in which they are the predominant host for ticks. In our study, we found B. lusitaniae in skin biopsy specimens and ticks that fed on green lizards. These findings implicate this species of lizard in the transmission cycle of B. lusitaniae. The competence of other lizard species that feed ticks should be also investigated. The low prevalence of B. lusitaniae in questing ticks, however, indicates that the ecology of B. lusitaniae in endemic foci of central Europe is more complex. Further studies that analyze the circulation of B. burgdorferi s.l. among a broader spectrum of host species should be undertaken.

## References

[1] Wang G, van Dam AP, Schwartz I, Dankert J. Molecular typing of Borrelia burgdorferi sensu lato: taxonomic, epidemiological, and clinical implications. Clin Microbiol Rev. 1999;12:633–53. 10515907 10.1128/cmr.12.4.633 PMC88929

[2] Masuzawa T, Takada N, Kudeken M, Fukui T, Yano Y, Ishiguro F, Borrelia sinica sp. nov., a Lyme disease–related Borrelia species isolated in China. Int J Syst Evol Microbiol. 2001;51:1817–24. 10.1099/00207713-51-5-1817 11594614

[3] Richter D, Schlee DB, Allgower R, Matuschka FR. Relationships of a novel Lyme disease spirochete, Borrelia spielmani sp. nov., with its hosts in Central Europe. Appl Environ Microbiol. 2004;70:6414–9. 10.1128/AEM.70.11.6414-6419.2004 15528500 PMC525186

[4] van Dam AP, Kuiper H, Vos K, Widjojokusumo A, de Jongh BM, Spanjaard L, Different genospecies of Borrelia burgdorferi are associated with distinct clinical manifestations of Lyme borreliosis. Clin Infect Dis. 1993;17:708–17. 10.1093/clinids/17.4.708 7903558

[5] Wang G, van Dam AP, Dankert J. Phenotypic and genetic characterization of a novel Borrelia burgdorferi sensu lato isolate from a patient with Lyme borreliosis. J Clin Microbiol. 1999;37:3025–8. 10449497 10.1128/jcm.37.9.3025-3028.1999 PMC85444

[6] Foldvari G, Farkas R, Lakos A. Borrelia spielmanii erythema migrans, Hungary. Emerg Infect Dis. 2005;11:1794–5. 16422006 10.3201/eid1111.050542 PMC3367353

[7] Collares-Pereira M, Couceiro S, Franca I, Kurtenbach K, Schafer SM, Vitorino L, First isolation of Borrelia lusitaniae from a human patient. J Clin Microbiol. 2004;42:1316–8. 10.1128/JCM.42.3.1316-1318.2004 15004107 PMC356816

[8] Diza E, Papa A, Vezyri E, Tsounis S, Milonas I, Antoniadis A. Borrelia valaisiana in cerebrospinal fluid. Emerg Infect Dis. 2004;10:1692–3. 15503409 10.3201/eid1009.030439 PMC3320289

[9] Hanincova K, Schafer SM, Etti S, Sewell HS, Taragelova V, Ziak D, Association of Borrelia afzelii with rodents in Europe. Parasitology. 2003;126:11–20. 10.1017/S0031182002002548 12613759

[10] Hanincova K, Taragelova V, Koci J, Schafer SM, Hails R, Ullmann AJ, Association of Borrelia garinii and B. valaisiana with songbirds in Slovakia. Appl Environ Microbiol. 2003;69:2825–30. 10.1128/AEM.69.5.2825-2830.2003 12732554 PMC154513

[11] Majlath I, Majlathova V. Green lizard (Lacerta viridis) as host for ectoparasites. Proceedings of abstracts and papers of the 5th International Conference on Ticks and Tick-Borne Pathogens. Neuchatel, Switzerland; 2005 29 Aug–2 Sep. Abstract 24.

[12] Tomassone L, Bertolotti L, Tramuta C, Nebbia P, Amore G, Ambrogi C, Bacterial tick-borne pathogens in ticks and vertebrate hosts in Tuscany (Italy). Proceedings of abstracts and papers of the 5th International Conference on Ticks and Tick-Borne Pathogens.Neuchatel, Switzerland; 2005 29 Aug–2 Sep:220–2.

[13] Younsi H, Postic D, Baranton G, Bouattour A. High prevalence of Borrelia lusitaniae in Ixodes ricinus ticks in Tunisia. Eur J Epidemiol. 2001;17:53–6. 10.1023/A:1010928731281 11523576

[14] Clark K, Hendricks A, Burge D. Molecular identification and analysis of Borrelia burgdorferi sensu lato in lizards in the southeastern United States. Appl Environ Microbiol. 2005;71:2616–25. 10.1128/AEM.71.5.2616-2625.2005 15870353 PMC1087528

[15] Wright SA, Lane RS, Clover JR. Infestation of the southern alligator lizard (Squamata: Anguidae) by Ixodes pacificus (Acari: Ixodidae) and its susceptibility to Borrelia burgdorferi. J Med Entomol. 1998;35:1044–9. 9835700 10.1093/jmedent/35.6.1044

[16] Lane RS, Loye JE. Lyme disease in California: interrelationship of Ixodes pacificus (Acari: Ixodidae), the western fence lizard (Sceloporus occidentalis), and Borrelia burgdorferi. J Med Entomol. 1989;26:272–8. 2769705 10.1093/jmedent/26.4.272

[17] Levin M, Levine JF, Yang S, Howard P, Apperson CS. Reservoir competence of the southeastern five-lined skink (Eumeces inexpectatus) and the green anole (Anolis carolinensis) for Borrelia burgdorferi. Am J Trop Med Hyg. 1996;54:92–7. 8651379 10.4269/ajtmh.1996.54.92

[18] Guy EC, Stanek G. Detection of Borrelia burgdorferi in patients with Lyme disease by the polymerase chain reaction. J Clin Pathol. 1991;44:610–1. 10.1136/jcp.44.7.610 1856296 PMC496808

[19] Black WC, Roehrdanz RL. Mitochondrial gene order is not conserved in arthropods: prostriate and metastriate tick mitochondrial genomes. Mol Biol Evol. 1998;15:1772–85. 9866211 10.1093/oxfordjournals.molbev.a025903

[20] Derdakova M, Beati L, Pet'ko B, Stanko M, Fish D. Genetic variability within Borrelia burgdorferi sensu lato genospecies established by PCR-single-strand conformation polymorphism analysis of the rrfA-rrlB intergenic spacer in Ixodes ricinus ticks from the Czech Republic. Appl Environ Microbiol. 2003;69:509–16. 10.1128/AEM.69.1.509-516.2003 12514035 PMC152394

[21] Fukunaga M, Hamase A, Okada K, Nakao M. Borrelia tanukii sp. nov. and Borrelia turdae sp. nov. found from ixodid ticks in Japan: rapid species identification by 16S rRNA gene-targeted PCR analysis. Microbiol Immunol. 1996;40:877–81. 8985944 10.1111/j.1348-0421.1996.tb01154.x

[22] Altschul SF, Madden TL, Schaffer AA, Zhang J, Zhang Z, Miller W, Gapped BLAST and PSI-BLAST: a new generation of protein database search programs. Nucleic Acids Res. 1997;25:3389–402. 10.1093/nar/25.17.3389 9254694 PMC146917

[23] Thompson JD, Higgins DG, Gibson TJ. CLUSTAL W: improving the sensitivity of progressive multiple sequence alignment through sequence weighting, positions-specific gap penalties and weight matrix choice. Nucleic Acids Res. 1994;22:4673–80. 10.1093/nar/22.22.4673 7984417 PMC308517

[24] Mather TN, Wilson ML, Moore SI, Ribeiro JM, Spielman A. Comparing the relative potential of rodents as reservoirs of the Lyme disease spirochete (Borrelia burgdorferi). Am J Epidemiol. 1989;130:143–50. 2787105 10.1093/oxfordjournals.aje.a115306

[25] Gern L, Rais O. Efficient transmission of Borrelia burgdorferi between cofeeding Ixodes ricinus ticks (Acari: Ixodidae). J Med Entomol. 1996;33:189–92. 8906929 10.1093/jmedent/33.1.189

[26] Ogden NH, Nuttall PA, Randolph SE. Natural Lyme disease cycles maintained via sheep by co-feeding ticks. Parasitology. 1997;115:591–9. 10.1017/S0031182097001868 9488870

[27] Randolph SE, Gern L, Nuttall PA. Co-feeding ticks: epidemiological significance for tick-borne pathogen transmission. Parasitol Today. 1996;12:472–9. 10.1016/S0169-4758(96)10072-7 15275266

[28] Piesman J, Donahue JG, Mather TN, Spielman A. Transovarially acquired Lyme disease spirochetes (Borrelia burgdorferi) in field-collected larval Ixodes dammini (Acari: Ixodidae). J Med Entomol. 1986;23:219. 3701806 10.1093/jmedent/23.2.219

[29] Kurtenbach K, De Michelis S, Etti S, Schafer SM, Sewell HS, Brade V, Host association of Borrelia burgdorferi sensu lato-the key role of host complement. Trends Microbiol. 2002;10:74–9. 10.1016/S0966-842X(01)02298-3 11827808

[30] Gern L, Hu CM, Kocianova E, Vyrostekova V, Rehacek J. Genetic diversity of Borrelia burgdorferi sensu lato isolates obtained from Ixodes ricinus ticks collected in Slovakia. Eur J Epidemiol. 1999;15:665–9. 10.1023/A:1007660430664 10543358

[31] Hubalek Z, Halouzka J. Prevalence rates of Borrelia burgdorferi sensu lato in host-seeking Ixodes ricinus ticks in Europe. Parasitol Res. 1998;84:167–72. 10.1007/s004360050378 9521004

[32] Hubalek Z, Halouzka J. Distribution of Borrelia burgdorferi sensu lato genomic groups in Europe, a review. Eur J Epidemiol. 1997;13:951–7. 10.1023/A:1007426304900 9476827

[33] Matuschka FR, Eiffert H, Ohlenbusch A, Richter D, Schein E, Spielman A. Transmission of the agent of Lyme disease on a subtropical island. Trop Med Parasitol. 1994;45:39–44. 8066380

[34] De Michelis S, Sewell HS, Collares-Pereira M, Santos-Reis M, Schouls LM, Benes V, Genetic diversity of Borrelia burgdorferi sensu lato in ticks from mainland Portugal. J Clin Microbiol. 2000;38:2128–33. 10834965 10.1128/jcm.38.6.2128-2133.2000 PMC86744

[35] Le Fleche A, Postic D, Girardet K, Peter O, Baranton G. Characterization of Borrelia lusitaniae sp. nov. by 16S ribosomal DNA sequence analysis. Int J Syst Bacteriol. 1997;47:921–5. 10.1099/00207713-47-4-921 9336887

[36] Wodecka B, Skotarczak B. First isolation of Borrelia lusitaniae DNA from Ixodes ricinus ticks in Poland. Scand J Infect Dis. 2005;37:27–34. 10.1080/00365540410026059 15764187

[37] Poupon M-A, Lommano E, Humair PF, Douet V, Rais O, Schaad M, Prevalence of Borrelia burgdorferi sensu lato in ticks collected from migratory birds in Switzerland. Appl Environ Microbiol. 2006;72:976–9. 10.1128/AEM.72.1.976-979.2006 16391149 PMC1352204

[38] Guner ES, Hashimoto N, Takada N, Kaneda K, Imai Y, Masuzawa T. First isolation and characterization of Borrelia burgdorferi sensu lato strains from Ixodes ricinus ticks in Turkey. J Med Microbiol. 2003;52:807–13.Clark K, Hendricks A, Burge D. Molecular identification and analysis of Borrelia burgdorferi sensu lato in lizards in the southeastern United States. Appl Environ Microbiol. 2005;71:2616–25. 12909659 10.1099/jmm.0.05205-0

